# Ruptured Abdominal Aortic Aneurysm in a Patient with Congenital Fused Pelvic Kidney: A Case Report of Emergency Endovascular Treatment

**DOI:** 10.1055/s-0038-1636992

**Published:** 2018-07-27

**Authors:** Claudio Bianchini Massoni, Matteo Azzarone, Danilo Barbarisi, Paolo Perini, Antonio Freyrie

**Affiliations:** 1 Vascular Surgery - Department of Surgical Sciences, Azienda Ospedaliero-Universitaria di Parma, Parma, Italy

**Keywords:** fused pelvic kidney, abdominal aortic aneurysm, aortic rupture, endovascular procedures

## Abstract

A 90-year-old male developed acute onset of abdominal and lumbar pain due to the rupture of an 11-cm abdominal aortic aneurysm. A congenital fused pelvic kidney perfused by three renal arteries arising from iliac axes was detected. In an emergent setting, an aorto-uni-iliac endograft was deployed through right femoral surgical access with occlusion of the upper right renal artery. An occluder device was placed in the common iliac artery above the renal artery through left femoral access. A femorofemoral crossover bypass completed the procedure. The patient developed acute renal failure, with no dialysis necessity. One-month computed tomography angiography showed procedure success.

## Introduction

Congenital renal abnormalities occur in 3.3 to 11% of the population 1 and include alterations in the number, shape, and/or site of the organ. 2 In patients with abdominal aortic aneurysm (AAA), the incidence of congenital renal variants is not well known. In a case series over a 20-year period, AAAs with congenital kidney anomalies were seen in 25 patients. 3

In this population, the number and site of origin of renal arteries are extremely variable and, for this reason, the treatment of AAA is more complex. Assessment of renal arteries (dimension, stenosis, and thrombus) and renal function (creatinine, serum urea level, and glomerular filtration rate) is mandatory for optimal planning and to obtain technical and clinical success after any type of procedure (surgical, endovascular, and hybrid).

Management of ruptured AAA is challenging and perioperative mortality after emergency open or endovascular treatment reaches 53%. 4

The aim of this article is to report a case of emergency endovascular treatment of a ruptured AAA with congenital fused pelvic kidney (CFPK).

## Case Presentation

**Fig. 1:**
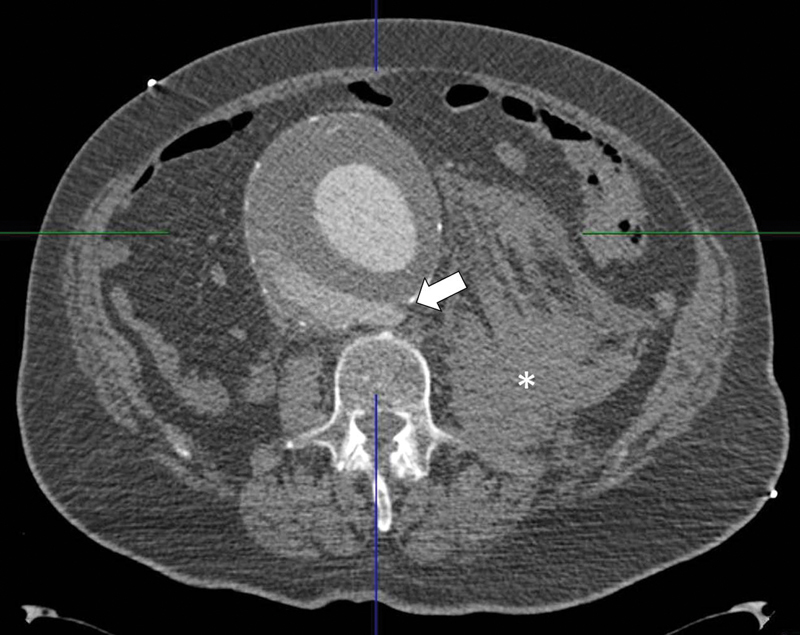
Computed tomography angiogram showing a voluminous abdominal aortic aneurysm (11.5 cm in diameter) with rupture of posterior aortic wall (white arrow) and intra-abdominal hematoma (white asterisk) on the left retroperitoneal region.

**Fig. 2:**
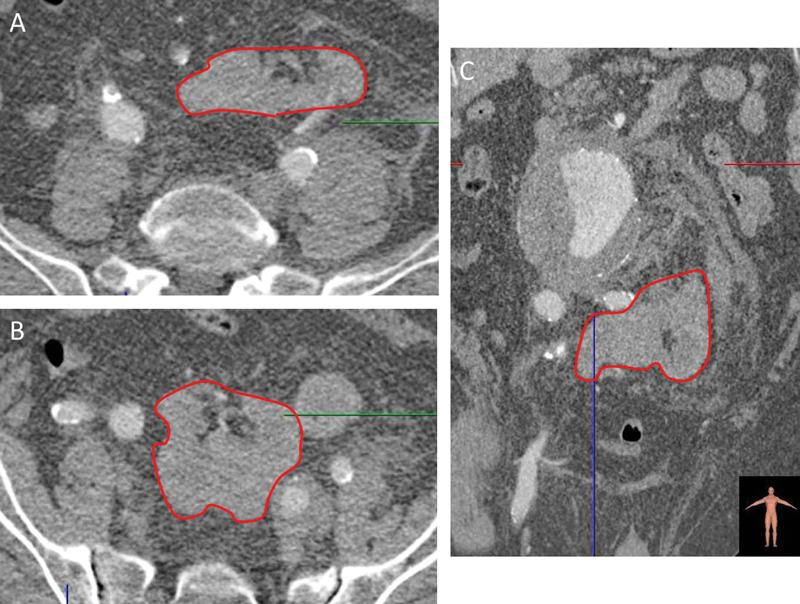
Computed tomography angiogram imaging of congenital fused pelvic kidney or pancake kidney. ( **A and B** ) axial images; red line: renal parenchyma. ( **C** ) Multiplanar reconstruction; red line: renal parenchyma.

**Fig. 3:**
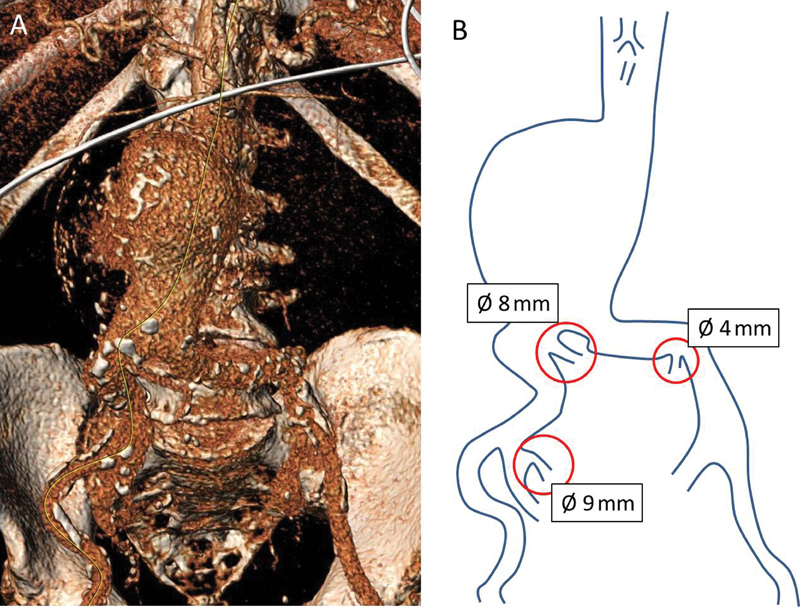
( **A** ) Volume rendering reconstruction of preoperative computed tomography angiogram (anteroposterior view). ( **B** ) Aortic outline and origin of renal arteries.

A 90-year-old male presented to the emergency department complaining of severe abdominal and back pain. The symptoms began 3 hours earlier. The patient was severely obese and under chronic pharmacological therapy for arterial hypertension and coronary artery disease (previous coronary artery bypass graft). The abdominal computed tomography angiogram (CTA) showed an 11-cm AAA with a retroperitoneal hematoma and a posterior wall rupture ( Fig. 1 ). The aortic rupture was partially contained by the vertebral bodies and retroperitoneum. A unique lobulated renal mass was detected in pelvic region (CFPK or pancake kidney 2 ) ( Fig. 2 ). Three renal arteries were detected arising from both iliac axes: one (8 mm in diameter) from the proximal portion of the right common iliac artery, the second (9 mm in diameter) from the right hypogastric artery, and the third (4 mm in diameter) from the distal segment of the left common iliac artery ( Fig. 3 ). The proximal neck (between the superior mesenteric artery and the AAA) was 20 mm in length and 23 to 24 mm in diameter with no intraluminal thrombus or wall calcification. The patient was conscious and hemodynamically stable (systolic blood pressure: 90–100 mm Hg). Considering the presence of a favorable proximal aortic neck, the partial hemodynamic stability, the age, and the obesity, an endovascular treatment was preferred. Only aorto-uni-iliac endografts were available for emergency procedures.

Preoperative planning was performed using 3mensio Vascular (Pie Medical Imaging) during patient transport to the operating theater and anesthetic induction.

**Fig. 4:**
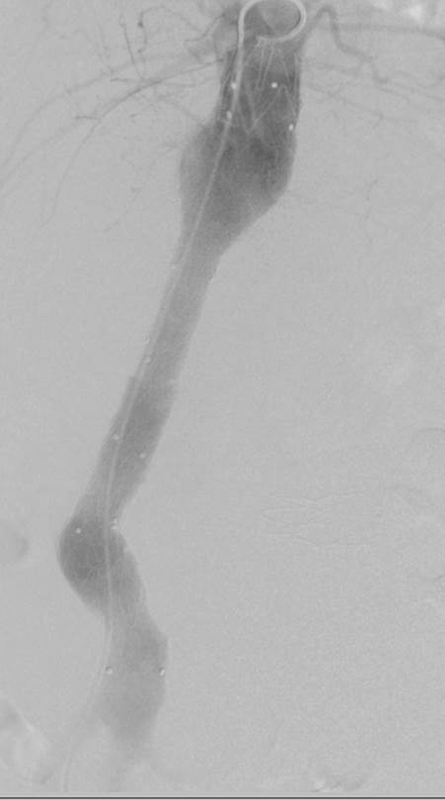
Anteroposterior digital subtraction angiogram after deployment of aortouniiliac endograft, two iliac extensions (through right femoral access) and occluder (through left femoral access); no endoleaks are detected.

Under general anesthesia, inguinal longitudinal cut-down was performed bilaterally and the common femoral arteries were isolated. An Endurant aorto-uni-iliac endograft (Medtronic Endovascular) was introduced through the right access. The C-arm was rotated at 50°-left anterior oblique and the preoperative angiogram was performed. The endograft was deployed below the origin of superior mesenteric artery, using the inframesenteric nondilated aorta as proximal sealing zone (∼20 mm in length). Two Endurant endograft extensions (Medtronic) were necessary to obtain sufficient sealing at the right common iliac artery with the occlusion of the upper renal artery. Through the left access, a Talent occluder was deployed in the proximal left common iliac artery, above the origin of left renal artery. The completion angiogram showed patency of the endografts and no endoleak from left iliac axis or renal or lumbar arteries ( Fig. 4 ). The procedure was completed with an extra-anatomical femorofemoral suprapubic bypass graft (8 mm-Silver Dacron). Operative procedure time was 220 minutes and 120cc of contrast agent was injected (iomeprol, Iomeron 300; Bracco).

**Fig. 5:**
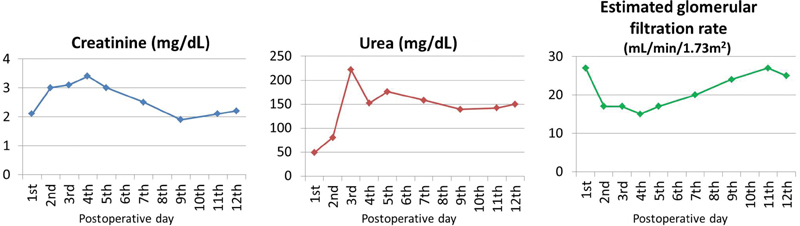
Serum levels of creatinine, urea, and estimated glomerular filtration rate (according to the Chronic Kidney Disease Epidemiology Collaboration Eq. 5 ) during hospitalization.

Ventilator support was removed after 24 hours and the abdominal and lumbar pain disappeared. Acute renal failure developed ( Fig. 5 5 ) but no dialysis was necessary. The patient returned to the ward on the 9th postoperative day and started mobilization. The postoperative duplex ultrasound showed endograft patency with no endoleak. Renal function gradually improved ( Fig. 5 ) and the patient was discharged on the 12th postoperative day.

**Fig. 6:**
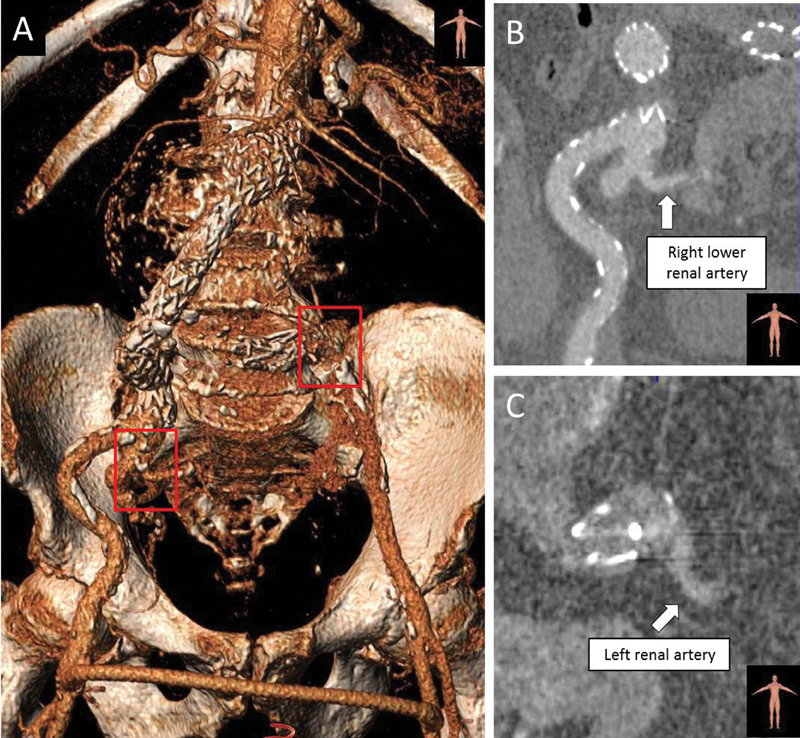
One-month computed tomography angiogram. ( **A** ) Volume rendering shows patency of celiac trunk, superior mesenteric artery, endograft, and femorofemoral crossover bypass. ( **B** ) Patency of lower right renal artery (arising from right hypogastric artery) (white arrow). ( **C** ) Patency of renal artery arising from left common iliac artery, below occluder device (white arrow).

At 1 month, the renal function remained mildly altered (creatinine serum level: 1.9 mg/dL). After intravenous hydration, the abdominal CTA showed patency of the aorto-uni-iliac endograft and femorofemoral bypass graft, sac shrinkage, and no evidence of endoleaks. The pelvic kidney presented no ischemic lesions and was perfused by two renal arteries (lower right and left) ( Fig. 6 ).

## Discussion

Congenital pelvic kidney (CPK) is defined as the presence of one ectopic kidney low lying in the abdomen. 2 This variant has been reported to be present in 1 to 2,200 to 1 in 3,000 individuals. 6

Congenital fused pelvic kidney, also called “pancake kidney,” is a rare congenital anatomic variant consisting of unique lobulated pelvic renal mass of dual parenchymatous systems. 2 During autopsies, this variant is observed in 1 out 22,000 cases. 7

Considering patients who underwent major aortic surgery, ∼0.18% presented a CPK. 8 Thus, the association between AAA and CFPK is extremely rare.

These two anatomical variants are often associated with multiple aberrant renal arteries arising from the aortic bifurcation and iliac arteries. AAA treatment is technically challenging, and has a higher risk of kidney failure.

In the current case, endovascular treatment was preferred because of the advanced age and the severe obesity of the patient.

No bifurcated endografts were available in our center for procedures in an urgent/emergent setting, so aorto-uni-iliac endografting and femorofemoral bypass were obligatory choices. Considering the sites of origin of the three renal arteries, the coverage of upper right renal artery was inevitable to obtain an adequate distal sealing zone. Also in case of left-side deployment of the aorto-uni-iliac endograft, the upper right renal artery would have been excluded by the occluder device deployment in the right common iliac artery.

To reduce the risk of persistent type 2 endoleak, a sac embolization may be intraoperatively performed. This adjunctive procedure was not executed to reduce time of intervention and was not considered strictly necessary. Indeed, no patent lumbar arteries had been detected at preoperatory CTA, and the upper right renal artery arises from a nondilated segment making unlikely a back flow into the aneurysmal sac.

In case of renal anomalies, assessment of preoperative CTA is essential to define their number, origin, and size. In the literature, sacrifice of accessory renal arteries with diameter < 3 mm is considered noninfluential on renal function. 9 In the current case, we excluded an 8-mm renal artery, sparing the other two renal arteries (9 mm and 4 mm in diameter). Transient renal function worsening was an expected event. Ultimately, no dialytic treatment was required. After 1 month, mild renal insufficiency persisted.

Several cases of treatment of AAA associated with CPK or CFPK have been reported in literature ( Table 1 ). 8 9 10 11 12 13 14 15 16 17 18 19 20 21 22 23 24 25 26 27 28 29 To our knowledge, the current case is the first reporting of the treatment of a ruptured AAA associated with the presence of CFPK.

**Table 1:** Cases of treated abdominal aortic aneurysm associated with pelvic kidney (congenital pelvic kidney or congenital fused pelvic kidney)

References	Year of publication	No. of patient	Congenital anomaly	Type of treatment	Type of aortic repair	Elective treatment
Ezzet et al 10	1977	1	Left CPK	Open	Dacron bifurcated graft	x
Hans and Robb 11	1984	1	Right CPK	Open	Dacron bifurcated graft	x
Hollis et al 12	1989	2	1. Right CPK	Open	1. Dacron bifurcated graft, 2 of 2 pelvic kidney arteries reimplanted to the main body of the graft	x
			2. Left CPK		2. Dacron tube graft, the lower of 2 pelvic kidney arteries included in the distal aortic anastomosis and the upper reimplanted in the left common iliac artery	x
Belcastro et al 13	1993	1	Right CPK	Open	Dacron tube graft	x
Schneider and Cronenwett 14	1993	1	Left CPK	Open	Dacron bifurcated graft, 1 of 1 pelvic kidney artery reimplanted to the right iliac limb of the graft	x
Glock et al 15	1997	1	Right CPK	Open	Dacron tube graft, 1 of 2 pelvic kidney arteries reimplanted, the other was included in the distal anastomosis	x
Kaplan et al 9	1999	1	CFPK	EV	Endovascular device (tube graft)	x
Rehrig et al 16	2001	1	Right CPK	Open	Dacron bifurcated graft, 1 of 1 pelvic kidney artery reimplanted	x
Faggioli et al 8	2003	3	NA	Open	Ectopic renal arteries reimplanted or included in the distal anastomosis	x
Murakami et al 17	2004	1	CFPK	Open	Dacron tube graft, 2 pelvic kidney arteries included in the distal anastomosis	x
Hanif et al 18	2005	1	Right CPK	Open	Dacron trifurcated graft, selective grafting of 1 of 1 pelvic kidney artery (9 mm graft)	x
Mandolfino et al 19	2005	1	Right CPK	Open	Dacron bifurcated graft, 2 of 2 pelvic kidney arteries reimplanted to the graft	x
Bui et al 20	2007	1	Left CPK	Open	Dacron bifurcated graft, 2 of 2 pelvic kidney arteries reimplanted to the graft	x
Coney et al 21	2008	1	Left CPK	Open	Dacron bifurcated graft, 2 of 2 pelvic kidney arteries reimplanted to the right iliac limb of the graft	x
Marone et al 22	2008	4	1. Right CPK	Open	1. Dacron tube graft, 1 of 1 pelvic kidney artery included distal anastomosis	x
			2. Left CPK	Open	2. Dacron tube graft, 1 of 1 pelvic kidney artery reimplanted to the graft	x
			3. Right CPK	Open	3. Dacron bifurcated graft, 1 of 1 pelvic kidney artery reimplanted to the main body of the graft	x
			4. Left CPK	Open	4. Dacron bifurcated graft, 1 of 2 pelvic kidney arteries reimplanted to the main body of the graft, the other was included in the distal anastomosis	x
Morales and Greenberg 23	2009	1	Left CPK	EV	Endovascular custom-made device	x
Makris et al 24	2011	1	CFPK	Open	Dacron tube graft, 1 of 2 pelvic kidney arteries included in the distal anastomosis (double shunt technique to prevent kidney ischemia)	x
Spear et al 25	2012	1	Left CPK	EV	Endovascular branched device	x
Jinnouchi et al 26	2012	1	Right CPK	Open	Dacron trifurcated graft, selective grafting of 1 of 1 pelvic kidney artery (8 mm graft)	x
Akashi et al 27	2012	1	Right CPK	Open	Dacron tube graft, 1 of 2 pelvic kidney arteries reimplanted to the graft	x
Malinowski et al 28	2014	1	CFPK	Hybrid	Hybrid technique: double Dacron graft was anastomized between two ectopic renal arteries and right EIA. Left IIA was embolized then EVAR was performed with Cook Zenith Alpha	x
Date et al 29	2015	1	Right CPK	Open	Dacron bifurcated graft, 3 of 3 pelvic kidney arteries reimplanted to the right iliac limb of the graft	x

Abbreviations: CFPK, cross-fused pelvic kidney; CPK, congenital pelvic kidney; EV, endovascular; EVAR, endovascular abdominal aortic aneurysm repair; NA, not available.

Surgical treatment of AAA in patients with pelvic kidney (CPK or CFPK) is more complex than in the general AAA population. Perfusion of the kidney must be maintained during aortic clamping and graft sewing. Renal arteries with diameter > 3mm should be reimplanted 9 and perfusion with cold Ringer solution is necessary in case of cross-clamp time > 40 minutes. 8

Total endovascular treatment has also been described. Morales and Greenberg 23 treated a patient with a type IV thoracoabdominal aortic aneurysm associated with a left CPK, deploying a custom-made fenestrated endograft. A fenestration near the distal bifurcation of the endograft permitted the perfusion of the CPK through a covered bridging stent. Also, a parallel stent graft technique has been described to perfuse an ectopic renal artery originating from the AAA in a patient with a crossover ectopic right kidney fused with the left kidney. 30 A Viabahn–Gore (W. L. Gore) endograft reinforced with balloon-expandable stents was deployed in a parallel fashion with an Endurant II–Medtronic endograft iliac limb.

Hybrid treatment has also been reported. In a case of CFPK with renal arteries arising from the AAA, a double iliac-renal bypass was primarily sutured and an aorto-bifurcated endograft was deployed to exclude the aneurysm. 28

In conclusion, in case of CPK or CFPK, endovascular, open surgical, and hybrid treatments of AAA have been described in literature and are potentially feasible. The most suitable treatment must be chosen according to the patient's clinical condition and arterial anatomy. Our case demonstrates that accurate preoperative planning is vital to achieve technical success and to preserve renal function in this complex subset of patients.
